# Formation of functional, extended bile canaliculi, and increased bile acid production in sandwich-cultured human cryopreserved hepatocytes using commercially available culture medium

**DOI:** 10.1007/s00204-024-03757-8

**Published:** 2024-05-16

**Authors:** Shinichiro Horiuchi, Yukie Kuroda, Ryota Oyafuso, Yuji Komizu, Kazuya Maeda, Seiichi Ishida

**Affiliations:** 1 Division of Pharmacology, National Institute of Health Sciences, Kawasaki, Japan; 2 Graduate School of Engineering, Department of Life Science, Sojo University, Kumamoto, Japan; 3 Laboratory of Pharmaceutics, School of Pharmacy, Kitasato University, Tokyo, Japan; 4 Laboratory of Molecular Pharmacokinetics, Graduate School of Pharmaceutical Sciences, The University of Tokyo, Tokyo, Japan

**Keywords:** Bile canaliculi, Bile acid, Cholestasis, Biliary excretion evaluation, Human cryopreserve hepatocyte, Sandwich-culture

## Abstract

Drug-induced cholestasis results in drug discontinuation and market withdrawal, and the prediction of cholestasis risk is critical in the early stages of drug development. Animal tests and membrane vesicle assay are currently being conducted to assess the risk of cholestasis in the preclinical stage. However, these methods have drawbacks, such as species differences with humans and difficulties in evaluating the effects of drug metabolism and other transporters, implying the need for a cholestasis risk assessment system using human hepatocytes. However, human hepatocytes hardly form functional, extended bile canaliculi, a requirement for cholestasis risk assessment. We previously established a culture protocol for functional, extended bile canaliculi formation in human iPSC-derived hepatocytes. In this study, we modified this culture protocol to support the formation of functional, extended bile canaliculi in human cryopreserved hepatocytes (cryoheps). The production of bile acids, which induces bile canaliculi extension, increased time-dependently during bile canaliculi formation using this protocol, suggesting that increased bile acid production may be involved in the extended bile canaliculi formation. We have also shown that our culture protocol can be applied to cryoheps from multiple donors and that bile canaliculi can be formed stably among different culture batches. Furthermore, this protocol enables long-term maintenance of bile canaliculi and scaling down to culture in 96-well plates. We expect our culture protocol to be a breakthrough for in vitro cholestasis risk assessment.

**Supplementary Information:**

The online version contains supplementary material available at 10.1007/s00204-024-03757-8.

## Introduction

The liver, the metabolic site for several drugs, is at high risk of being damaged by drugs. Moreover, drug-induced liver injury (DILI) is one of the primary reasons for drug withdrawal from the market (Onakpoya et al. 2016). Based on the mechanism of action, DILI is classified into the following three types: hepatocellular, cholestatic, or mixed injury. Among 1,676 DILI cases that were reported in Japan between 1997 and 2006, 40% had cholestatic or mixed cholestatic hepatotoxicity (Takikawa et al. 2009). Cholestatic liver injury, including mixed hepatocellular/cholestasis, is the most severe form of DILI. The exacerbation risk in patients with DILI is increased by cholestasis with a case fatality rate of 10–50% (Navarro and Senior 2006). Some drugs that cause cholestatic or mixed hepatocellular-cholestatic liver injury, such as troglitazone and bosentan, have been withdrawn from the market (John 2000; Mathieu 2015). Therefore, accurate evaluation of the cholestasis risk in the early stages of drug development is important.

The causes underlying drug-induced cholestasis are manifold (Padda et al. 2011; Kolarić et al. 2019; Vinken 2018). Functional inhibition of biliary efflux transporters by drugs is one of the most frequent causes of drug-induced cholestasis (Fattinger et al. 2001; Stieger et al. 2000; Navarro and Senior 2006). Cholestatic drugs disrupt biliary efflux transporter function through direct or indirect inhibition, including modulation of transporter localization or expression (Yang et al. 2013); for instance, troglitazone competitively inhibits the biliary efflux transporter, bile salt export pump (BSEP) (Funk et al. 2001). BSEP mediates bile acid transport from the cytoplasm to the bile canaliculi. Therefore, inhibition of BSEP induces liver injury through intracellular accumulation of bile acids. Animal experiments have been conducted in the preclinical stage but have limitations, such as species differences in bile acid composition and regulation or expression of biliary efflux transporters (Morgan et al. 2010). To overcome these limitations, the membrane vesicle assay (MVA) has been used to evaluate the DILI risk associated with the inhibition of biliary efflux transporter (Dawson et al. 2012; Morgan et al. 2013). However, MVA is insufficient for risk assessment of drug-induced cholestasis because it cannot consider the effects of drug metabolism and interactions with other transporters. This necessitates a cell-based assay, which can accurately evaluate the effects of drugs on biliary excretion in humans to detect drugs with cholestatic risk before clinical tests.

Bile canaliculi formation is required to evaluate biliary excretion, and sandwich culture is performed for bile canaliculi formation (Deharde et al. 2016). In sandwich-cultured hepatocytes, basolateral and biliary transporters are appropriately localized via polarization. However, extended bile canaliculi, which are required for a cholestasis toxicity test and biliary efflux evaluation, are not formed in the sandwich culture of cryopreserved human hepatocytes (cryoheps) using a general hepatocyte maintenance medium (Supplementary Fig. 1). We previously established a culture protocol for functional, extended bile canaliculi formation in human iPSC-derived hepatocytes (hiPSC-Heps) using a commercially available medium (Horiuchi et al. 2022). In addition, we have confirmed that the bile canaliculi formed using this protocol have the biliary excretion ability via the biliary efflux transporter. Moreover, due to the limited availability of cells from the same donor, a protocol that can stably form extended bile canaliculi in cells from different donors is required when cryoheps are used to assess the cholestasis risk. Therefore, in the present study, based on Horiuchi et al. (2022), we examined the culture conditions for forming functional, extended bile canaliculi in cryoheps to establish a culture protocol for canaliculi formation in cryoheps. We also examined whether the culture protocol for bile canaliculi formation can be applied to cells from different donors. Furthermore, we examined whether the cells could be exposed to drugs for a longer duration in culture to mimic chronic liver toxicity and attempted to scale down culture conditions for high throughput screening.

Hepatocyte polarization, which relates to bile canaliculi formation, correlates with changes in energy metabolism (Gissen and Arias 2015; Treyer and Müsch 2013). In addition, the activation of 5' adenosine monophosphate-activated protein kinase (AMPK), which is an energy sensor (Hardie 2011), promotes bile canaliculi formation (Fu et al. 2010). Bile acids that regulate lipid and sugar metabolism (Radun and Trauner 2021) promote bile canaliculi formation via AMPK activation (Fu et al. 2011). Therefore, we examined the changes in bile acid production during culture and reported that bile acid production increased during the formation of bile canaliculi.

## Materials and methods

### Chemicals

Fluorescein diacetate (Sigma-Aldrich, MO, USA), 5-(and-6)-carboxy-2',7'-dichloro-fluorescein diacetate (FUJIFILM, Tokyo, Japan), and N-(24-[7-(4-N,N-dimethylaminosulfonyl-2,1,3-benzoxadiazole)]amino-3α,7α,12α-trihydroxy-27-nor-5β-cholestan-26-oyl)-2'-aminoethane-sulfonate (Genomembrane Co., Ltd. Yokohama, Japan) were used as model substrates to characterize transporter-mediated biliary excretion. MK571 (Cayman Chemical, MI, USA), cyclosporine A (FUJIFILM), and 2',5'-dideoxyadenosine (Sigma-Aldrich) were used as inhibitors of Multidrug Resistance-Associated Protein 2 (MRP2), BSEP, and AMPK, respectively.

### Culture for bile canaliculi formation in a 24-well plate

**Fig. 1:**
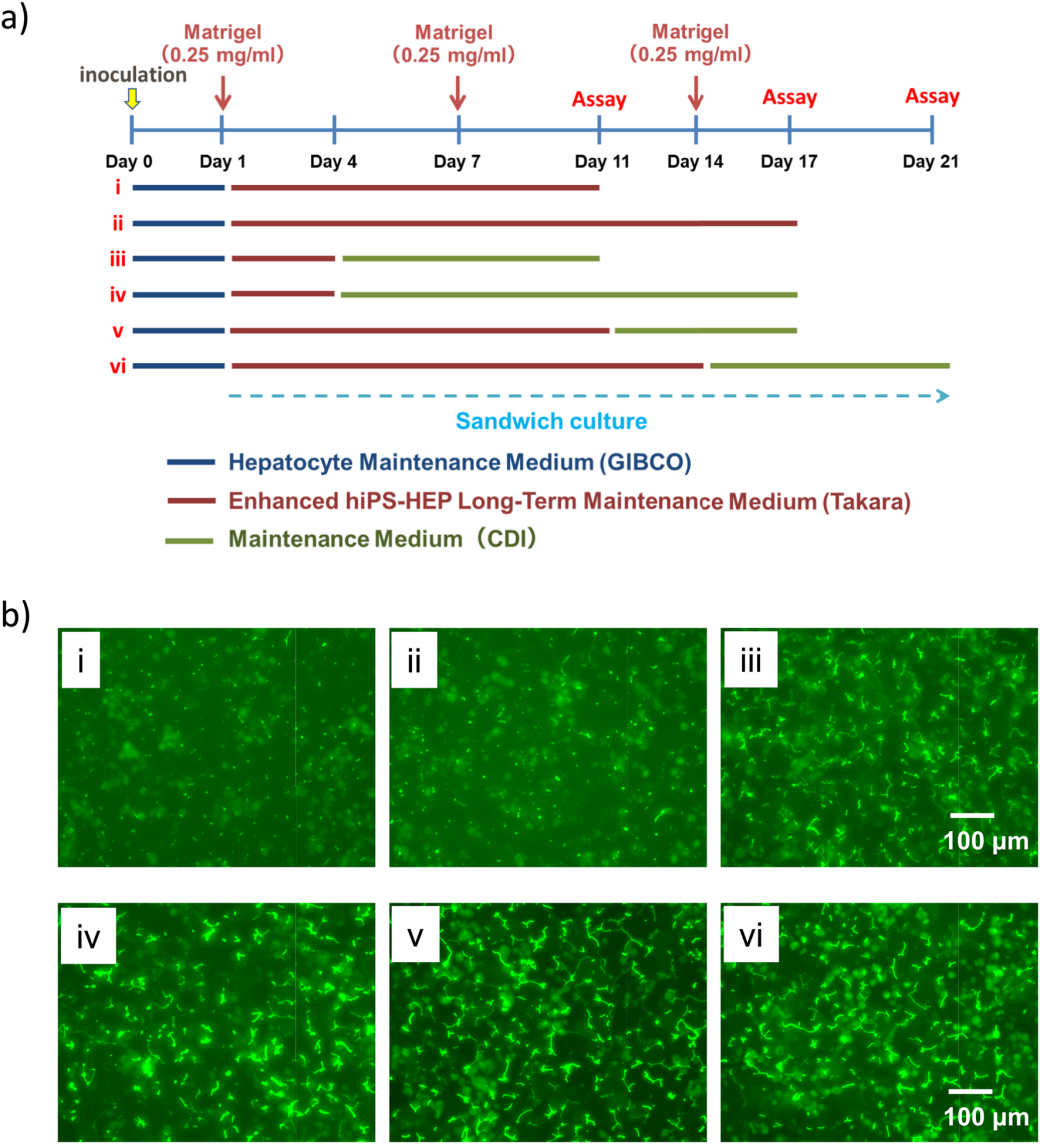
Examination of extended bile canaliculi formation conditions in cryoheps. **a** Schematic diagram of culture conditions; cryoheps (lot: HC10-10) were cultured under each condition. **b** Bile canaliculi observed using CDFDA at the endpoint of each culture condition. Fluorescence images show CDF, which was accumulated into the bile canaliculi

Cryoheps from 4 donors (lot: HC2-50, 3–3, 3–30, 10–10) were obtained from XenoTech (Lenexa, KS, USA). After thawing the cells using OptiThaw (XenoTech), they were suspended in an OptiPlate (XenoTech) at 7.2 × 10^5^ cells/mL. Subsequently, 500 μL of the cell suspension was seeded on a collagen-coated 24-well plate (Corning, NY, USA). After 4–5 h, the medium was replaced with 500 µL of Hepatocyte Maintenance medium (HM medium, Supplementary Table 1). On the next day, the medium was replaced with 800 μL of Cellartis® Enhanced hiPS-HEP Long-Term Maintenance (LTM) Medium (Takara, Otsu, Japan) supplemented with Matrigel (Corning), and a sandwich culture was initiated. Thereafter, the time point of changing the medium to iCell hepatocyte Maintenance medium (CM medium, Supplementary Table 2) for bile canaliculi formation was examined under the conditions shown in Fig. 1a. During this period, the medium was replaced every 2–3 d, and Matrigel was overlaid on Day 7 and Day 14. The amount of medium was 800 μL (2 d apart) or 1200 μL (3 d apart) each.

### Culture for bile canaliculi formation in a 96-well plate

After thawing cells using OptiThaw, cells were suspended in an OptiPlate at 7.2 × 10 ^5^ cells/mL. Then, 85 μL of the cell suspension was seeded on a collagen-coated 96-well plate (Corning, NY, USA). The cell-seeded plate was flash-centrifuged at 300 X g at 23° C. After 4–5 h, the medium was replaced with 100 µL of HM medium. On the next day, the medium was replaced with 140 μL of LTM medium supplemented with Matrigel, and sandwich culture was started. Thereafter, the LTM medium was changed to CM medium on Day 11. During this period, the medium was changed every 2–3 d, and Matrigel was overlaid on Day 7 and 14. The volume of the medium was 140 μL (2 d apart) or 210 μL (3 d apart).

### Biliary efflux assay

The cells were incubated in a CO_2_ incubator (5% CO_2_, 37° C) for 10 min in CM medium supplemented with 4 μM 5-(and-6)-carboxy-2',7'-dichloro-fluorescein diacetate (CDFDA). After replacement with fresh medium, the cells were incubated in a CO_2_ incubator for 10 or 15 min to excrete 5-(and-6)-carboxy-2',7'-dichloro-fluorescein (CDF) into the bile canaliculi. The cells were incubated in a CO_2_ incubator for 30 min in CM medium supplemented with 40 μM tauro-nor-THC-24-DBD. After replacement with fresh medium, the cells were incubated in a CO_2_ incubator for 30 min to excrete tauro-nor-THC-24-DBD into the bile canaliculi. The fluorescence model substrate was imaged using BZ-9000 (KEYENCE, Osaka, Japan).

### Inhibition assay of biliary excretion

The cells were pre-incubated for 30 min in CM medium supplemented with MK571 (50 μM). Subsequently, the cells were incubated for 10 min in CM medium supplemented with 4 μM CDFDA and 50 μM MK571. Thereafter, the medium was changed to a maintenance medium supplemented with 50 μM MK571, and the cells were incubated for 10 min to excrete CDF into the bile canaliculi. The cells were pre-incubated for 30 min in CM medium supplemented with 10 μM cyclosporin A (CsA). After pre-incubation, the cells were incubated for 30 min in CM medium supplemented with 40 μM tauro-nor-THCA-24-DBD and 10 μM CsA. Thereafter, the medium was changed to CM medium supplemented with 10 μM CsA, and the cells were incubated for 30 min to excrete tauro-nor-THCA-24-DBD into the bile canaliculi. The fluorescence model substrate, which accumulated in bile canaliculi, was imaged using BZ-9000. Fluorescence images were captured at the same exposure time, with or without inhibitor.

### AMPK inhibition assay

Cryoheps were sandwich-cultured in LTM medium for 10 d from the next day of seeding and then in CM medium with or without 2',5'-dideoxyadenosine for 4 or 5 d, respectively. Bile canaliculi were observed via CDFDA assay or immunostaining. The area of each bile canaliculus stained with anti-BSEP was measured using ImageJ (https://imagej.nih.gov/ij/index.html), and the average value was calculated excluding ≤ 10 μm^2^ in each image.

### Immunostaining

Immunostaining for MRP2, BSEP, and actin filaments (F-actin) was performed as described previously (Horiuchi et al. 2022).

### Quantitative measurement of bile acids

Cells were collected with a cell scraper after adding Dulbecco S Phosphate-Buffered Saline (PBS: Sigma-Aldrich). The collected cell suspension was sonicated on ice using the Handy Sonic UK-20P (intensity 4, 30 s × 5, Tomy Seiko, Tokyo, Japan). After centrifugation (10,000 × g, 10 min, 4°°C), the supernatant was collected. Bile acid content in the supernatant was measured using the Total Bile Acid Assay Kit (Cell Biolabs Inc. San Diego, CA, USA), according to the manufacturer’s instructions. In addition, the 28 types of bile acids were measured using the LC/MS/MS (LC–MS-8060N, Shimadzu Co, Kyoto, Japan) Method Package for Bile Acids, at Shimadzu Techno-Research, Kyoto, Japan (https://www.shimadzu-techno.co.jp/annai/pha/h21.html). The cut-off value of each compound was a peak area of 3000; the peak area was normalized to the value on Day 16 as 1.

### Measurement of gene expression

Total RNA was isolated from the cells, and the expression of the genes listed in Supplementary Table 3 was measured using TaqMan real-time polymerase chain reaction (PCR), as described previously (Horiuchi et al. 2022). The expression of *CYPs* during extended bile canaliculi formation was compared with the maximum and minimum expression in 22 lots of cryoheps under vendor-recommended conditions (Horiuchi et al. 2023).

### Measurement of metabolic activity

The activities of CYPs during extended bile canaliculi formation were measured using liquid chromatography–tandem mass spectrometry (LC–MS/MS) as described previously (Horiuchi et al. 2022). The results were compared with the maximum and minimum expression in 8 lots of cryoheps under vendor-recommended conditions (Horiuchi et al. 2023).

### Statistical analysis

Bar graph values are expressed as mean ± SD. Significance tests for the effects of AMPK inhibitors on bile canaliculi formation were performed using a Student’s t-test in Excel. Values of *P* < 0.01 were considered statistically significant.

## Results

### Examination of culture condition for bile canaliculi formation

In hiPSC-Heps, functional, extended bile canaliculi could be formed using the following culture method: hiPSC-Heps were cultured in Cellartis® Enhanced hiPS-HEP LTM medium for 28 d, followed by sandwich-culture in CM medium (Horiuchi et al. 2022). Therefore, in the present study, we attempted to form functional, extended bile canaliculi in cryoheps via sandwich culture using LTM and CM medium. Cryoheps were cultured under the six conditions shown in Fig. 1a, and bile canaliculi formation was observed using CDFDA. We observed that extended bile canaliculi were formed on the whole well surface by culturing in CM medium, following sandwich-culture in LTM medium (Fig. 1a, Supplementary Fig. 2). In particular, bile canaliculi were extremely extended by culture in the CM medium after more than 10 d of sandwich culture in the LTM medium. Therefore, in subsequent experiments, the medium was switched from LTM to CM medium on Day 11 to facilitate optimal conditions for the extended bile canaliculi formation.

### Evaluation of bile excretory capacity of the bile canaliculi

**Fig. 2:**
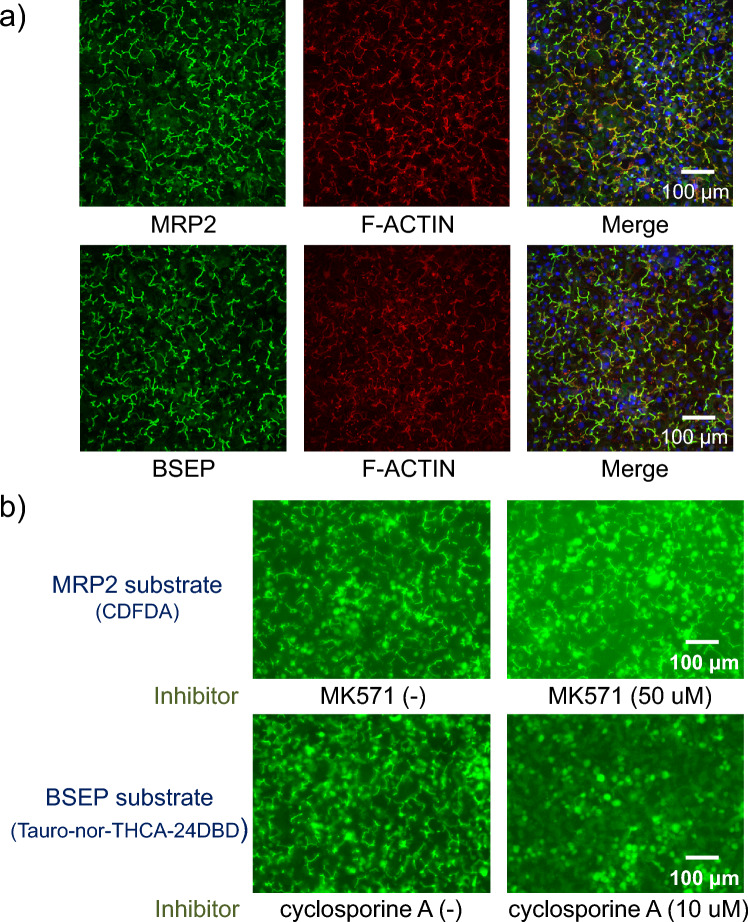
Evaluation of biliary excretion ability via MRP2 or BSEP; cryoheps (lot: HC10-10) were sandwich-cultured in Long-Term medium for 10 d from the next day of seeding and then in CDI maintenance medium for 5 d. At the endpoint, immunostaining and biliary excretion evaluation were performed. **a** Immunostaining image. MRP2 or BSEP (green). Actin filaments (red), representing bile canaliculi structures. Nucleus (blue). **b** Excretion of a fluorescence model substrate (FDA, tauro-nor-THC-24DBD) into the bile canaliculi and the effect of biliary efflux transporter inhibitors. Fluorescence images of MK571- or cyclosporin A-treated cells, which were treated with biliary efflux transporter inhibitors (right) or untreated (left), showing CDF or Tauro-nor-THCA-24-DBD

Biliary excretion function in bile canaliculi formed via culture for 16 d was evaluated. The fluorescence image of F-ACTIN, which lines the bile canaliculi (Javitt 2020), showed the formation of extended bile canaliculi structures. (Fig. 2a). In the immunofluorescence analysis, signals of MRP2 and BSEP, which are biliary efflux transporters, overlapped with those of F-ACTIN, suggesting that MRP2 and BSEP were co-localized in the bile canaliculi structures (Fig. 2a). The evaluation of biliary excretion using CDFDA and Tauro-nor-THCA-24DBD, which are fluorescence model substrates for MRP2 and BSEP, respectively, showed the accumulation of these model substrates in the bile canaliculi (Fig. 2b). Furthermore, the intracellular fluorescence intensity was enhanced upon exposure to inhibitors of the respective biliary efflux transporters, indicating the inhibition of the excretion of these model substrates. These results suggest that the formed bile canaliculi had biliary efflux transporter-dependent bile excretion ability.

### Time course of cell morphology, expression of biliary uptake/efflux transporters, and Cytochrome P450

**Fig. 3:**
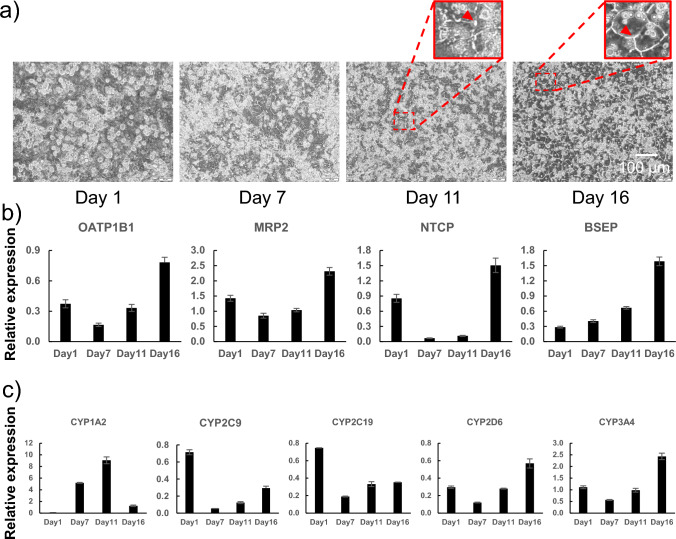
Time course of cell morphology, expression of biliary uptake/efflux transporters, and Cytochrome p450 (CYP); cryoheps (lot: HC3-30) were sandwich-cultured in Long-Term medium for 10 d from the next day of seeding and then in the CDI maintenance medium for 5 d. During the culture period, phase-contrast images were captured, and expression of biliary uptake/efflux transporters was measured. **a** Time course of the phase-contrast image. Time course of the expression of **b** biliary uptake/efflux transporters and **c** major CYPs. Bars show the relative expression levels. Pooled RNA from the human liver was used for the standard curve, and the expression level was set as one. The relative expression level was calculated using the equation of the line for the standard curve. Data are presented as means ± SD (*n* = 3)

The observation of the bile canaliculi using CDFDA showed that the extended bile canaliculi were barely observable in LTM medium cultures for 10 d (Fig. 1b–i) but became observable in subsequent CM medium cultures. (Fig. 1b–v). Observation with a phase-contrast microscope also showed that bile canaliculi-like structures were extended in CM medium cultures after Day 11 (Fig. 3a). When the bile canaliculi were extended in CM medium cultures, the expression of the uptake transporters *OATP1B1*, *NTCP*, and efflux transporters *MRP2* and *BSEP* increased (Fig. 3b). The expression of the uptake/efflux transporter on Day 16, when the extended bile canaliculi were formed, was higher than that on Day 1, and was equal to or higher than that in human liver (relative expression level = 1). Drug metabolism activity decreases with long-term culture (Bell et al. 2018). Therefore, we measured the expression of major Cytochrome P450s (CYPs) over time during the culture. *CYP1A2* expression was high on Day 7 and 11 during culture in the LTM medium (Fig. 3c). The expression of other *CYPs* decreased on Day 7 after switching to the LTM medium and then increased as the culture period lengthened. In addition, the expression of *CYP1A2*, *CYP2D6*, and *CYP3A4* on Day 16, when extended bile canaliculi were formed, was higher than that on Day 1. The expression levels of *CYP2C9* and *CYP2C19* on Day 16 were approximately half of that on Day 1 and did not differ by an order of magnitude. Consequently, no remarkable decrease in *CYP* expression was observed with long-term culture.

### Comparison of bile canaliculi formation and Cytochrome P450 expression among cells from multiple donors

**Fig. 4:**
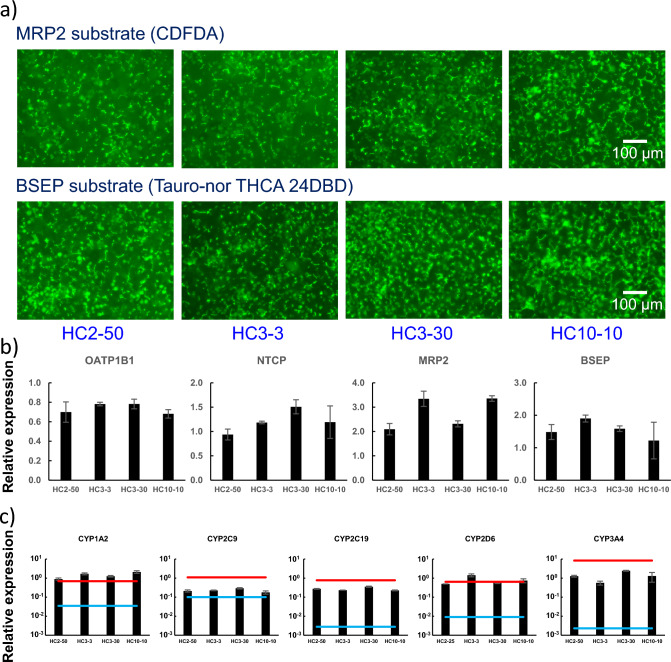
Excretion of fluorescence model substrates into bile canaliculi and gene expression of bile uptake/efflux transporters during bile canaliculi formation in different lots of cryoheps. Four lots of cryoheps were sandwich-cultured in Long-Term medium for 10 d from the next day of seeding and then in the CDI maintenance medium for 5 d. At the endpoint, the excretion of the fluorescence model substrate into the bile canaliculi was observed and the expression of biliary uptake/efflux transporters was measured. **a** Excretion into bile canaliculi of FDA or tauro-nor-THC-24DBD. Fluorescence images show FDA or tauro-nor-THC-24DBD, which was accumulated into bile canaliculi. **b** Expression of biliary uptake/efflux transporters. Bars shows the relative expression levels. Pooled RNA from the human liver was used for the standard curve, and the expression level was set as one. The relative expression level was calculated using the equation of the line for the standard curve. Data are presented as means ± SD (*n* = 3). **c** Expression of major CYPs. Bars show the relative expression levels. Pooled RNA from the human liver was used for the standard curve, and the expression level was set as one. The relative expression level was calculated using the equation of the line for the standard curve. Data are presented as means ± SD (*n* = 3). Red and blue lines show maximum and minimum values of the expression in 22 lots of cryoheps under vendor-recommended conditions (Horiuchi et al. 2023)

We have shown above that bile canaliculi formation culture for 16 d in one donor cryoheps can form functional, extended bile canaliculi and that the expression of *CYPs* during extended bile canaliculi formation is comparable to that on Day 1 of culture. Therefore, cryoheps from 4 donors were cultured, and the formed bile canaliculi and the expression of *CYPs* and biliary uptake/efflux transporters were compared between donors. Immunostaining results showed that extended bile canaliculi were formed in all the lots (supplementary Figs. 3 and 4). However, length and density of bile canaliculi were slightly different among the lots. Observation of the bile canaliculi using a fluorescence model substrate showed the accumulation of the fluorescence model substrate in the bile canaliculi of cells from all donors, suggesting that the biliary efflux transporter is functional (Fig. 4a). Expressions of bile uptake/efflux transporters were similar to or higher than that in the human liver (Fig. 4b). The expression of *CYPs* when the extended bile canaliculi were formed was similar to or higher than that of cryoheps cultured under vendor-recommended conditions (Fig. 4c). In addition, the activity of CYP1A, CYP2C19, CYP2D6, and CYP3A in lot HC3-30 cells was the same as that in cryoheps under vendor-recommended conditions (supplementary Fig. 5).

### Comparison of bile canaliculi formation between culture batches

The formed bile canaliculi were compared between culture batches to evaluate the reproducibility of the established culture protocol. Similar fluorescein images of bile canaliculi were observed in all batches (supplementary Fig. 6). This confirmed the reproducibility of the culture protocol for bile canaliculi formation.

### Stability of canalicular excretion ability

**Fig. 5:**
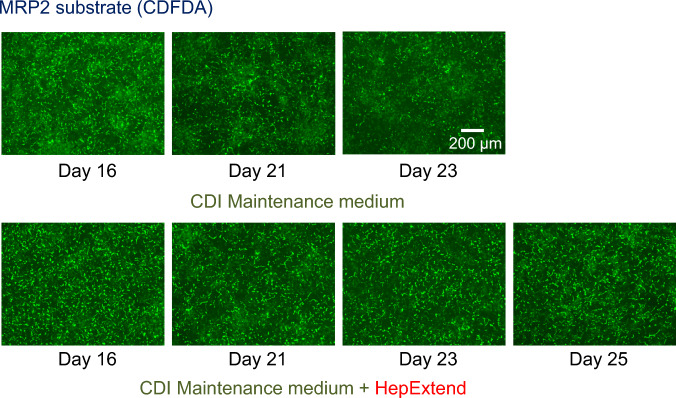
Stability of formed bile canaliculi; cryoheps (lot: HC10-10) were sandwich-cultured in Long-Term medium for 10 d from the next day of seeding and then in the CDI maintenance medium with or without HepExtend for 14. Bile canaliculi were observed using CDFDA from Day 16 to 25. Fluorescence images show CDF, which was accumulated into bile canaliculi

The period in which functional, extended bile canalicular excretion can be stably maintained was examined. When the cells were cultured in CM medium, the observed bile canaliculi decreased after 21 d. However, similar fluorescein images were observed up to Day 25 by adding HepExtend, which is a supplement for long-term culture, to CM medium (Fig. 5). These results suggest that functional, extended canalicular excretion is stably maintained for at least 9 d.

### Scale down of culture condition for high throughput screening

The culture protocol for the formation of functional, extended bile canaliculi on 96-well plates was examined. When the cells were seeded in a 96-well plate according to the protocol for seeding cells in a 24-well plate, the cell adhesion rate was low after 5 h of cell seeding, and the cells did not adhere to the well surface in certain regions on the Day 16 (supplementary Fig. 7a). Flash centrifugation was performed after cell seeding in order to improve the cell adhesion rate. As a result, cells adhered to the whole well surface, forming extended bile canaliculi (supplementary Figs. 7a and b). Moreover, CDF and Tauro-nor-THCA-24DBD were confirmed to be excreted into the bile canaliculi.

### Bile acid production and bile canaliculi formation

**Fig. 6:**
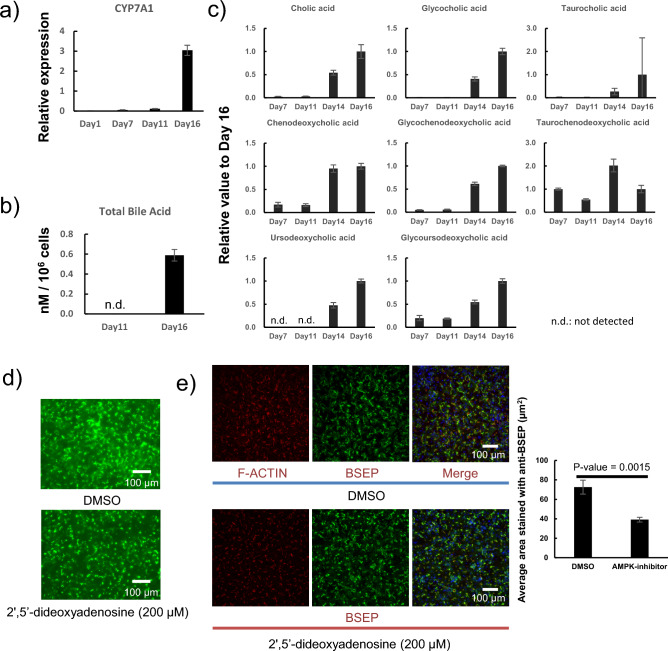
CYP7A1 expression and bile acid production during bile canaliculi formation; cryoheps (lot: HC3-30) were sandwich-cultured in Long-Term medium for 10 d from the next day of seeding and then in the CDI maintenance medium (CM medium) for 4 or 5 d. **a** Expression of CYP7A1, the rate-limiting enzyme for bile acid biosynthesis. Bars show the relative expression levels. Pooled RNA from the human liver was used for the standard curve, and the expression level was set as one. The relative expression level was calculated using the equation of the line for the standard curve. Data are presented as means ± SD (*n* = 3). **b** Total bile acid content in cells and bile canaliculi. **c** Relative value of liver-produced bile acids, and their glyco- or tauro-conjugates, detected by LC–MS/MS. Relative value was calculated with the value on Day 16 set as 1. 2',5'-Dideoxyadenosine was added during the 5 d culture on the CM medium, and the effect on the length of the bile canaliculi was observed. Bile canaliculi were observed using **d** CDFDA assay and **e** immunostaining. The bar graph shows the average area of each bile canaliculus stained by anti-BSEP in one image. Data are presented as means ± SD (*n* = 3)

Gene expression levels of *CYP7A1*, a rate-limiting enzyme in the synthesis of bile acid from cholesterol, were measured. *CYP7A1* expression markedly increased from Day 11 to 16, when the bile canaliculi extended rapidly (Fig. 6a). In addition, total bile acid levels were measured in cells and bile canaliculi and were below the detection limit on Day 11 but reached a sufficiently detectable level on Day 16 (Fig. 6b). When the 28 types of bile acids were evaluated using LC–MS/MS, 11 types of bile acids were detected, 8 of which were liver-produced bile acids (cholic acid, chenodeoxycholic acid, and ursodeoxycholic acid) and their glyco- or tauro-conjugates (glycocholic acid, taurocholic acid, chenodeoxycholic acid, taurochenodeoxycholic acid, and glycoursodeoxycholic acid). Concentrations of all liver-produced bile acids and their glyco- or tauro-conjugates increased after Day 14 (Fig. 6c, Supplementary Table 4). This result suggests that bile acid production increased from the Day 11 to Day 16 when the bile canaliculi extended rapidly. Taurocholate, one of the bile acids, activates AMPK and extends the bile canaliculi (Fu et al. 2011). Therefore, the effect on bile canaliculi formation was examined by adding 2',5'-dideoxyadenosine, an AMPK inhibitor, to the CM medium. This shortened the length of the bile canaliculi (Fig. 6d and e).

## Discussion

We have previously established a culture protocol to form functional, extended bile canaliculi in hiPS-Heps using a commercially available medium (Horiuchi et al. 2022). In the present study, we modified this protocol to form functional, extended bile canaliculi in cryoheps. Extended bile canaliculi could be formed on the whole well surface when cryoheps were sandwich-cultured in LTM medium from Day 1 to 11 and in CM medium for more than 5 d from Day 11. The formed bile canaliculi were confirmed to have a transporter-dependent biliary excretion ability. In addition, the expression of biliary uptake/efflux transporters during bile canaliculi formation was at the same or above the level in the human liver. These results suggest that our culture protocol may be applied to evaluate cholestatic toxicity and biliary excretion of pharmaceuticals and other compounds. Kitaguchi et al. (2023) applied our culture protocol to assess the biliary excretion of food compounds. They reported that the biliary excretion clearance of the compounds is closer to that in the human liver using our culture protocol compared to previous reports (Abe et al. 2008). However, it still differs from in vivo biliary excretion clearance, thereby warranting correction with a scaling factor for in vitro to in vivo extrapolation (IVIVE). We believe that an improvement in biliary excretion ability is needed to bring biliary excretion clearance even closer to that in vivo*.* Furthermore, cryoheps cultured using our protocol maintained functional bile canaliculi for 9 d, suggesting the possibility of applying it to risk assessment during repeated exposure.

A limitation of using cryoheps to evaluate cholestatic toxicity and biliary excretion is the limited supply of cells from the same donor. In contrast, hiPSC-Heps derived from the same donor can be stably supplied. In addition, no clear difference in the length and density of bile canaliculi formation was observed in hiPSC-Heps between culture batches (Horiuchi et al. 2022). Therefore, hiPSC-Heps may facilitate stable repeated experiments with cells from the same donor over several years. In the present study, we attempted to form bile canaliculi using 4 lots of cells from different donors and confirmed that extended bile canaliculi were formed in all lots and had biliary excretion ability. In addition, the expression of *CYPs* during bile canaliculi formation in all the lots was comparable to that of cryoheps under vendor-recommended conditions. These results suggest that our culture protocol can be used with cells from various donors. Furthermore, in the same lot, similar bile canaliculi were successfully formed using different culture batches, suggesting the possibility of a stable evaluation of cholestatic toxicity and biliary excretion. However, the density and length of bile canaliculi differed among lots. Therefore, future studies should examine the effects of inter-lot differences in the density and length of bile canaliculi on the evaluation of cholestatic toxicity and biliary excretion.

Several drugs show efficacy and toxicity through drug metabolism. Therefore, drug metabolism is an important consideration in cholestasis risk assessment. When bile canaliculi were formed using our culture protocol, the expression of major *CYPs* in all lots was comparable to that of cryoheps under vendor-recommended conditions. In addition, the activity of CYP1A, CYP2C19, CYP2D6, and CYP3A in lot HC3-30 cells was comparable to that of cryoheps under vendor-recommended conditions. Therefore, we believe that the cholestatic toxicity and biliary excretion via the metabolism of CYPs can be evaluated using our culture protocol. In a previous report, the expression of *CYP1A2* and *CYP2C9* and the activity of CYP1A, CYP2C9, and CYP2D6 in hiPCS-Heps during cilia canaliculi formation was lower than that in cryoheps under vendor-recommended conditions (Horiuchi et al. 2022). Therefore, the evaluation of cholestatic toxicity and biliary excretion using cryoheps is expected to better reflect metabolism than that using hiPSC-Heps.

In our culture protocol for bile canaliculi formation, the dot-shaped bile canaliculi transformed into extended bile canaliculi upon replacing the medium from LTM to CM medium. Concurrently, the expression of *CYP7A1*, a rate-limiting enzyme in bile acid synthesis, was markedly increased; bile acid production was also increased. In addition, the time-dependent increase of liver-produced bile acids and their conjugates after Day 14 was confirmed by measuring individual bile acids using LC/MS/MS. Accordingly, bile canaliculi extension was correlated with bile acid production. Bile acids are known as ligands for nuclear receptors, such as farnesoid X receptor (FXR) and G-protein coupled receptors, and act as signaling molecules to regulate lipid and glucose metabolism (Radun and Trauner 2021). During liver development and regeneration, hepatocyte polarization correlates with changes in energy metabolism (Gissen and Arias 2015; Treyer and Müsch 2013). AMPK, which monitors the AMP/ATP and ADP/ATP ratio in cells, is involved in bile canaliculi formation (Fu et al. 2010). Furthermore, taurocholate, which increased after Day 14, has also been reported to promote bile canaliculi network formation through AMPK activation (Fu et al. 2011). In our culture protocol, the addition of the AMPK inhibitor 2',5'-dideoxyadenosine from Day 11 to Day 16, when the bile canaliculi were extending, suppressed bile canaliculi extension. These results suggest that an increased production of bile acids may be involved in bile canaliculi extension in our culture protocol. Therefore, the identification of components that increase bile acid production in the CM medium may facilitate more efficient bile canaliculi formation. The bile canaliculi formed after replacement with CM medium tended to be longer in proportion to the culture period in the LTM medium. This trend has also been observed in hiPSC-Heps (Horiuchi et al. 2022). Accordingly, culture in the LTM medium may also be related to the extended bile canaliculi formation. In the future, we intend to identify the components involved in bile canaliculi formation by focusing on bile acid production and cell–cell adhesion. These examinations are expected to lead to further improvement of the medium for bile canaliculi formation and to the production of a medium consisting of known components. Takemura et al. have reported that drugs that inhibit the formation of bile canaliculi cause cholestasis (Takemura et al. 2016). The confirmation of the inhibition of bile canaliculi extension by 2',5'-dideoxyadenosine suggests the possibility of applying our culture protocol for screening drugs that induce cholestasis by inhibiting bile canaliculi formation. In addition, bile acids have been reported to be involved in the biliary excretion ability. Bile acid (chenodeoxycholic acid), an agonist of FXR, promotes biliary excretion by increasing BSEP expression (Nell et al. 2022). FXR activation leads to increased expression of MRP and BSEP (Kast et al. 2002; Zollner et al. 2003). The expression of MRP2 and BSEP and bile acid levels increased between Day 11 and Day 16, suggesting that bile acids promote biliary excretion by increasing the expression of biliary excretion transporters.

In this study, the results of the biliary efflux transporter or AMPK inhibitor assay suggest the possibility of applying our culture protocol to evaluate transporter- and bile canaliculi formation-dependent cholestasis. Our culture protocol for bile canaliculi formation can be replicated at any research institution because it uses commercially available cryoheps and culture medium. In addition, our culture protocol will enable the evaluation of multiple samples via scaling down to a 96-well plate. Therefore, we believe that our culture protocol can contribute to popularizing in vitro evaluation systems for cholestasis and biliary excretion. Furthermore, our culture protocol is expected to expand the measurement range and improve the accuracy of biliary excretion evaluation by increasing the amount of bile that can be accumulated through bile canaliculi extension. However, we believe that improving biliary uptake/excretion transporter activity is necessary to equate biliary excretion clearance and in vivo values. We expect that the publication of our culture protocol will be a breakthrough in the evaluation of cholestasis and biliary excretion. Moreover, we hope that our studies will help identify drugs that cause cholestasis before the preclinical stage, thereby reducing the number of animal studies and drug development costs.

### Supplementary Information

Below is the link to the electronic supplementary material.

Supplementary file1 (PDF 1419 KB)

## Data Availability

Data sharing not applicable to this article as no datasets were generated or analysed during the current study.
