# Supplementary material for: Formation of functional, extended bile canaliculi, and increased bile acid production in sandwich-cultured human cryopreserved hepatocytes using commercially available culture medium
Source: Arch Toxicol. 2024 May 16;98(8):2605–17. doi: 10.1007/s00204-024-03757-8 (PMC11272753; doi:10.1007/s00204-024-03757-8)
Supplement: Supplementary file 1 — Supplementary file1 (PDF 1419 KB) [file 204_2024_3757_MOESM1_ESM.pdf]

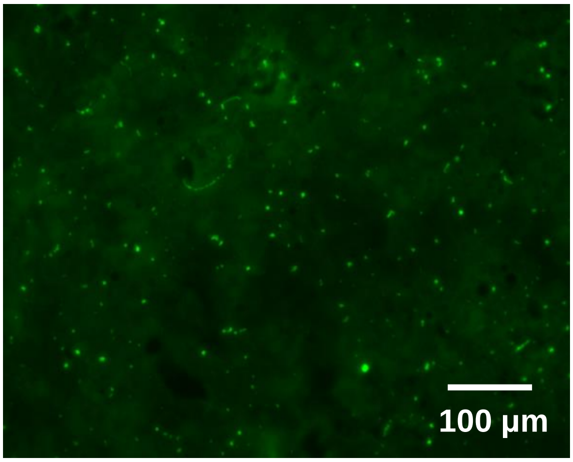

Supplementary figure 1.

Bile canaliculi formation in sandwich-culture using general hepatocyte culture medium. cryoheps (lot: HC10-10) were seeded on collagen coated 24-well plate and cultured in Hepatocyte Maintenance Medium for 24 hours. Then, the cells were overlaid with Matrigel and sandwich-cultured in Hepatocyte Maintenance Medium for 3 days. Bile canaliculi were observed using CDFDA. Fluorescence images show CDF, which was accumulated into bile canaliculi.

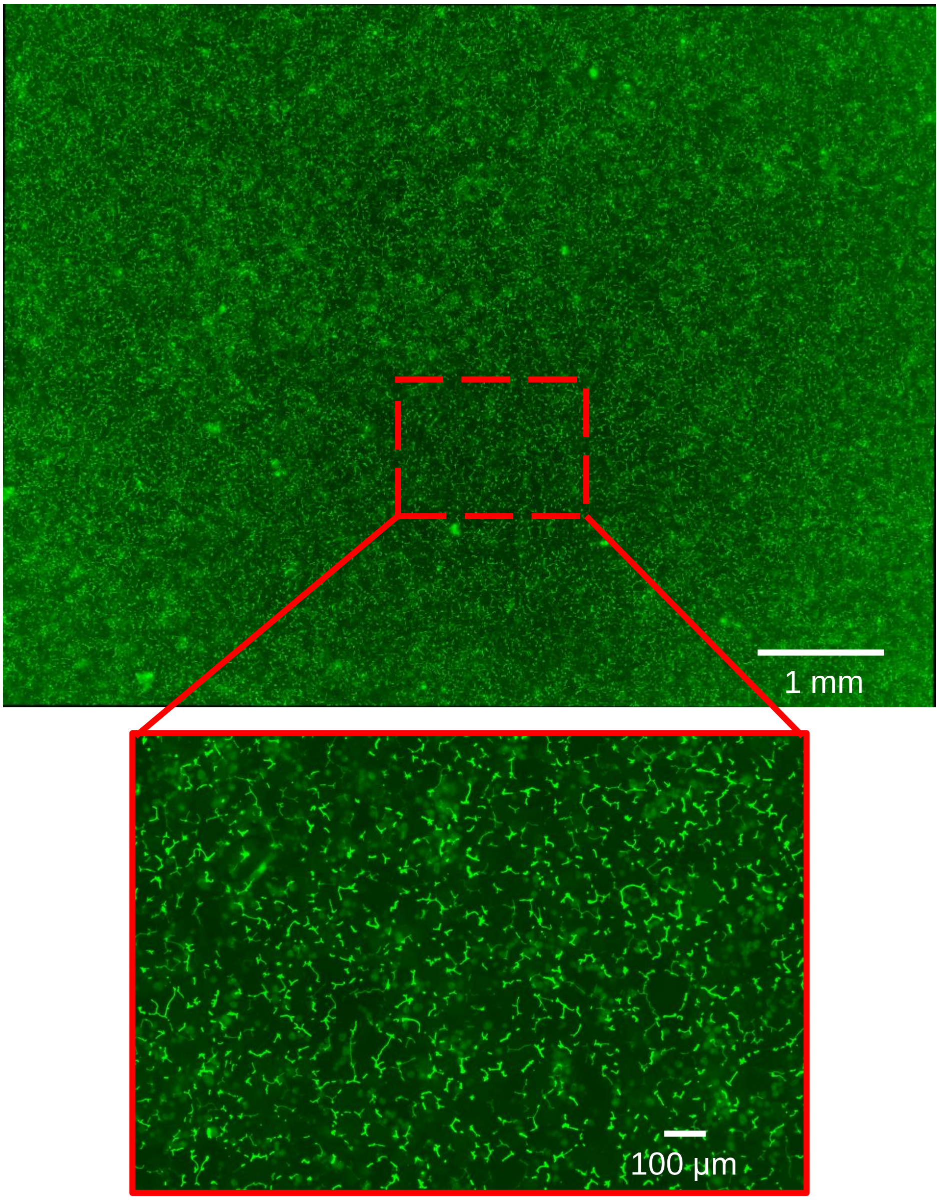

Supplementary figure 2.
Observation of bile canaliculi over a wide area. cryoheps (lot: HC10-10) were sandwich-cultured in Long Term medium for 10 days from the next day of seeding and then in CDI maintenance medium for 6 days. At the endpoint, the biliary efflux assay was performed in each batch culture using CDFDA. Fluorescence images show CDF accumulated in bile canaliculi.

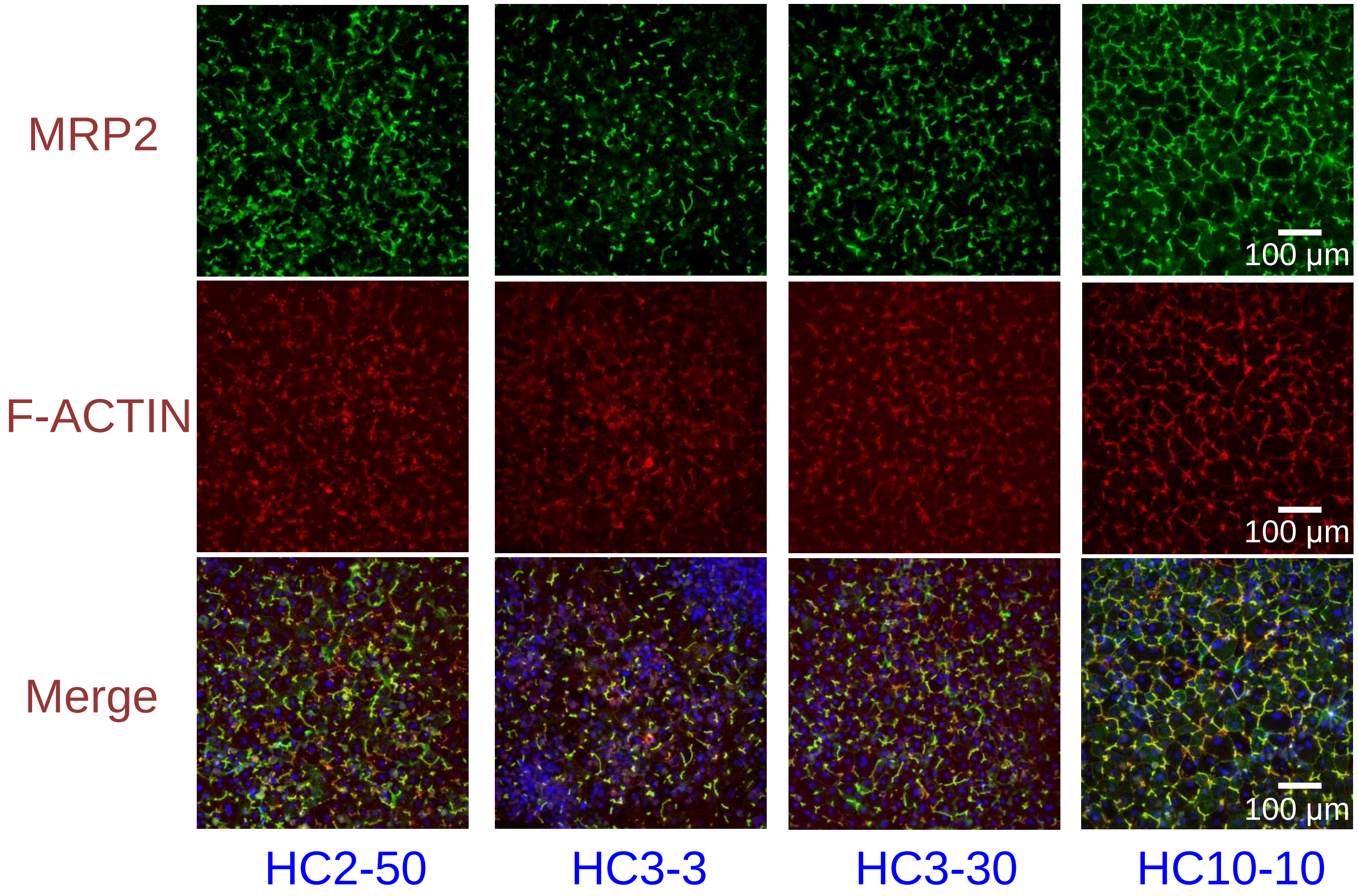

Supplementary figure 3.

Immunostaining image of biliary efflux transporters in four lots of cryoheps. The four lots of cryoheps were sandwich-cultured on Long Term medium for 10 days from the next day of seeding and then in maintenance medium for 5 days. At the endpoint, the cells were immunostained. MRP2 (green). Actin filaments which indicative of bile canaliculi structures (red). Nucleus (blue).

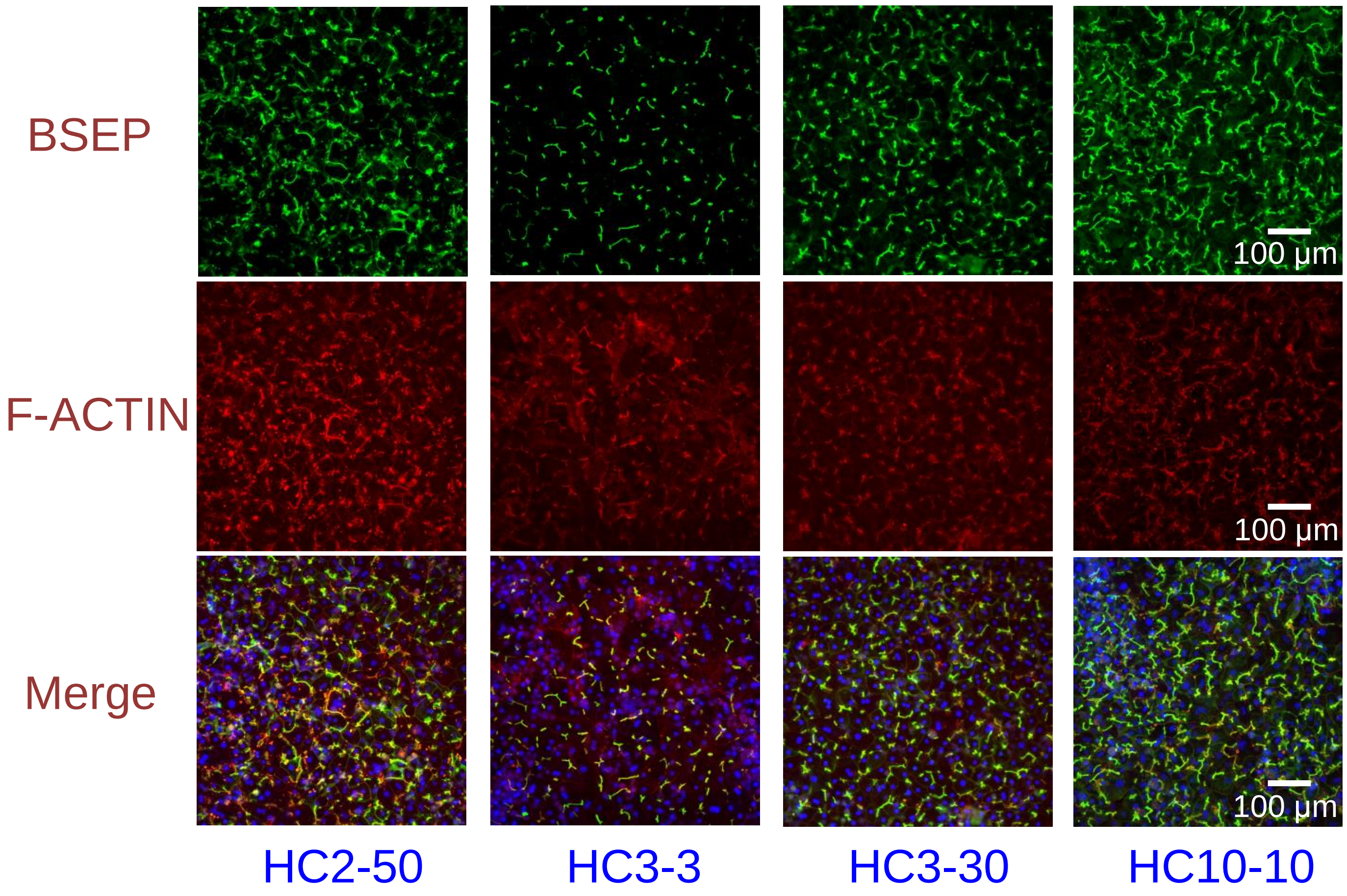

Supplementary figure 4.

Immunostaining image of biliary efflux transporters in four lots of cryoheps. The four lots of cryoheps were sandwich-cultured on Long Term medium for 10 days from the next day of seeding and then in maintenance medium for 5 days. At the endpoint, the cells were immunostained. BSEP (green). Actin filaments which indicative of bile canaliculi structures (red). Nucleus (blue).

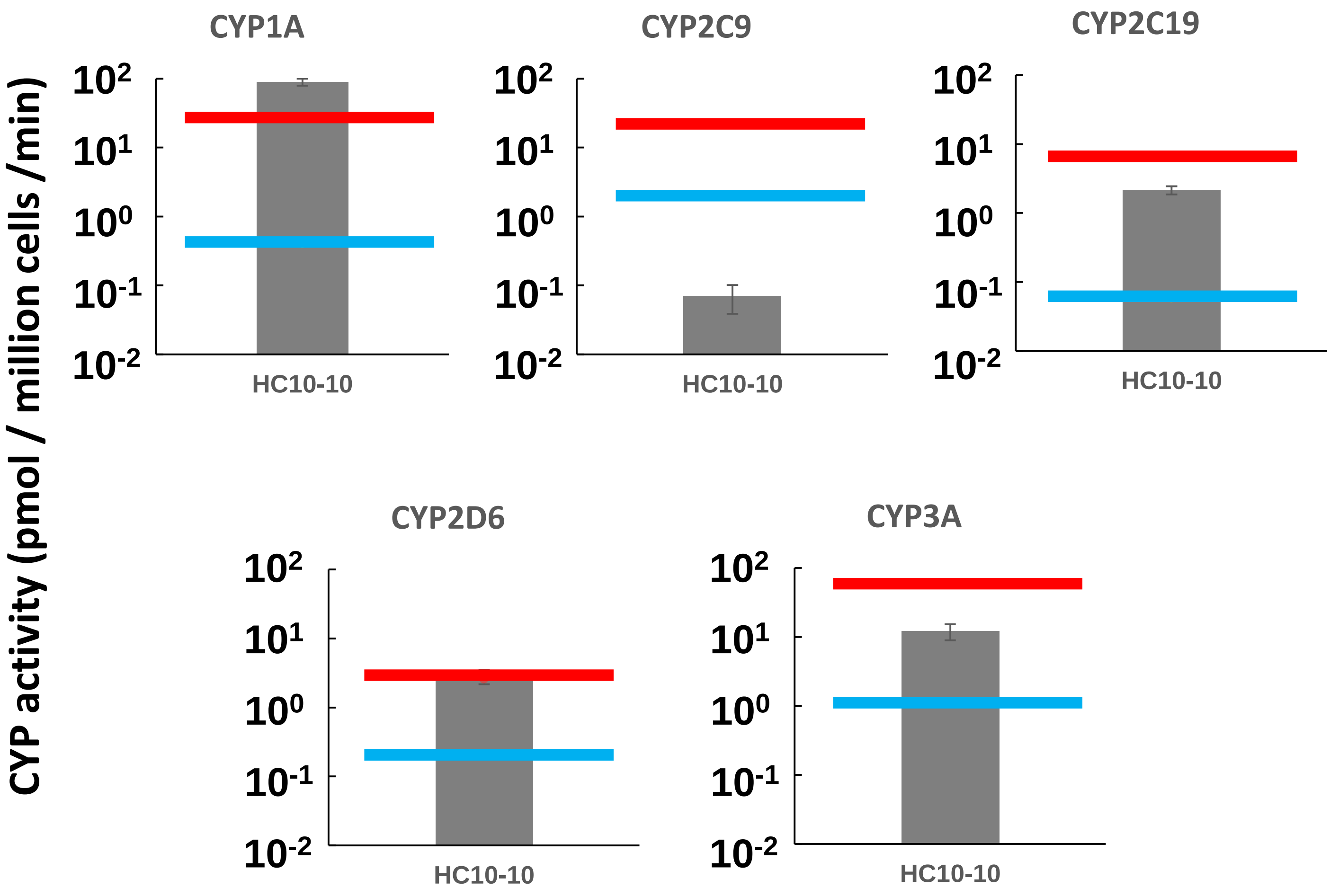

Supplementary figure 5.
Cytochrome P450 activity when the bile canaliculi were formed. cryoheps (lot: HC3-30) were sandwich-cultured in Long Term medium for 10 days from the next day of seeding and then in CDI maintenance medium for 5 days. At the endpoint, the metabolism test was performed. The red and blue line shows the maximum and minimum value of activity in 8 lots of cryoheps under vendor-recommended conditions (Horiuchi et al. 2023). Data are presented as means ± S.D. (n = 3).

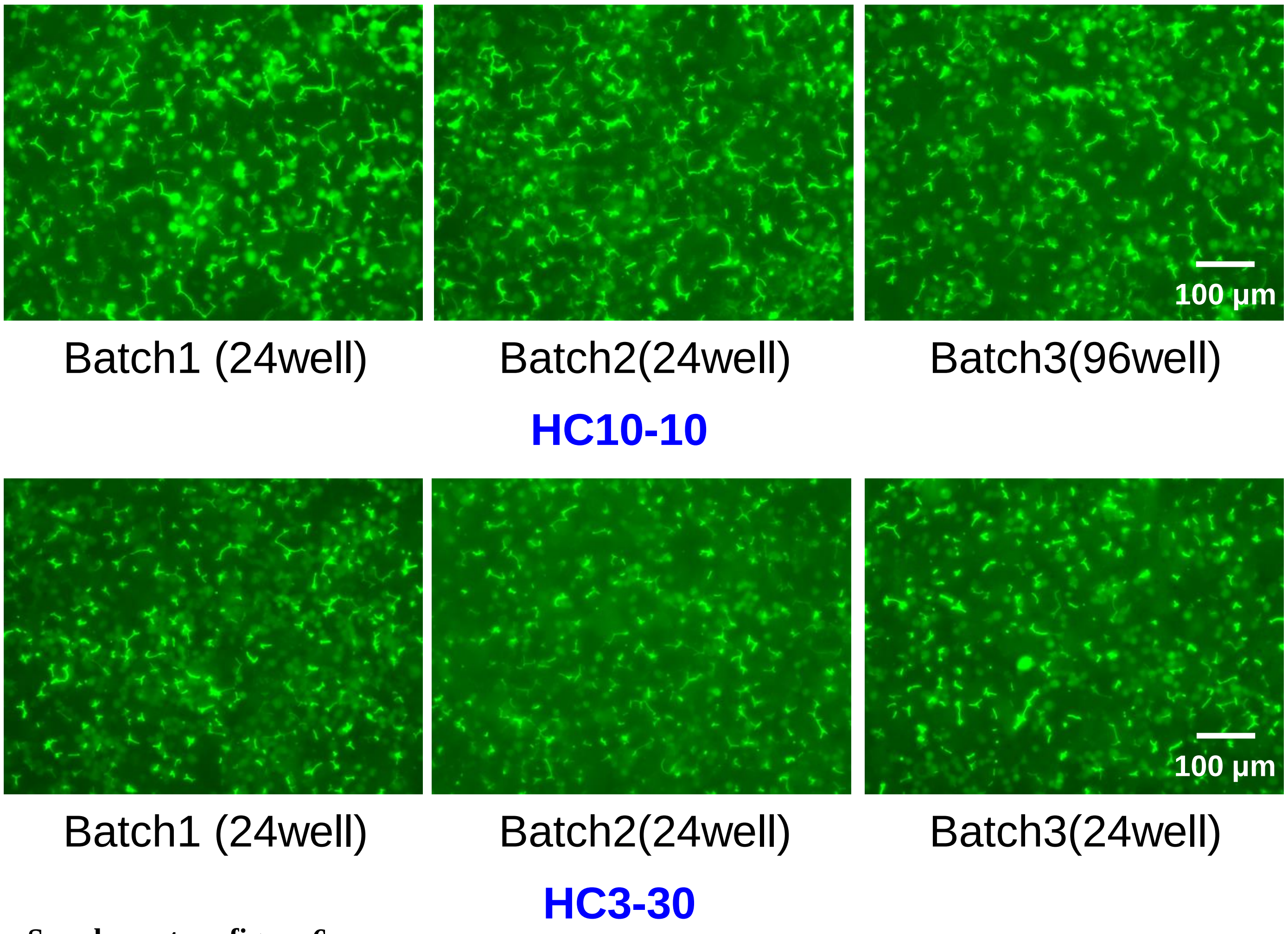

Supplementary figure 6.
Comparison of bile canaliculi formation between culture batches. cryoheps (lot: HC-30 or HC10-10) were sandwich-cultured in Long Term medium for 10 days from the next day of seeding and then in CDI maintenance medium for 5 days. At the endpoint, the biliary efflux assay was performed in each batch culture using CDFDA. Fluorescence images show CDF accumulated in bile canaliculi**.**

a)

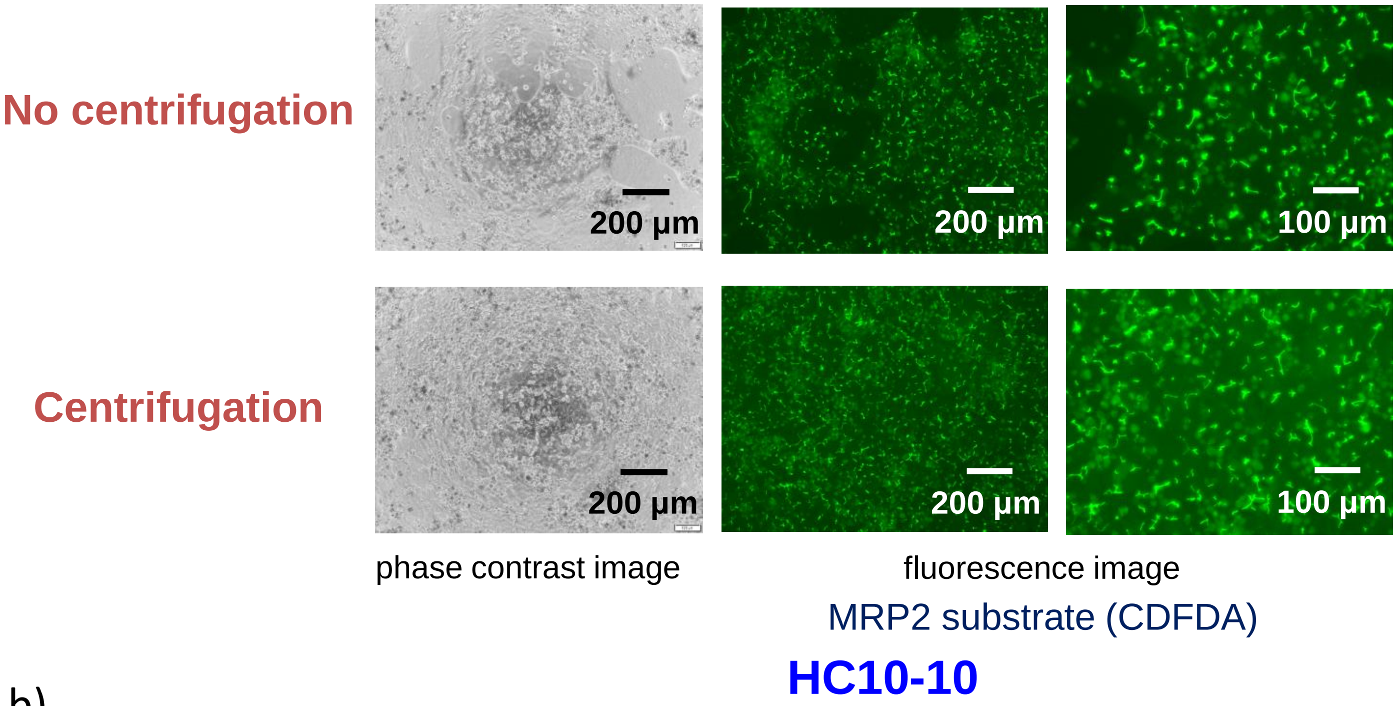

b)

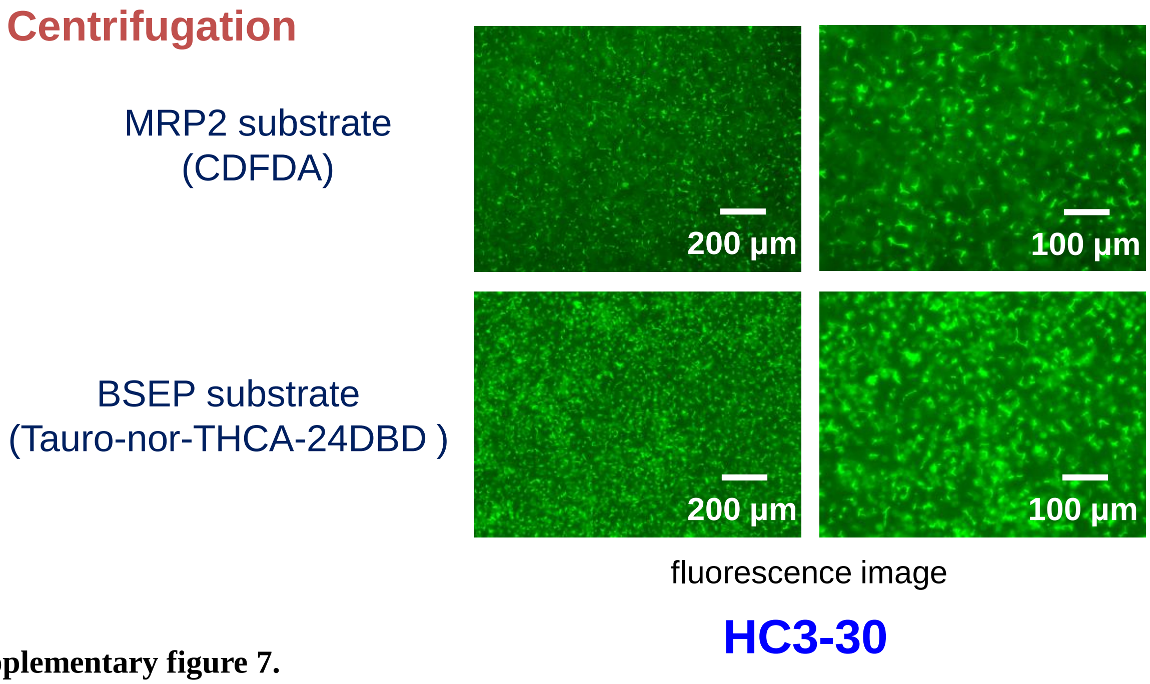

Supplementary figure 7.

Scale-down culture for bile canaliculi formation. cryoheps (lot: HC3-30) were sandwich-cultured in Long Term medium for 10 days from the day after seeding cells on a 96-well plate and in then CDI maintenance medium for 5 days. At the endpoint, the biliary efflux assay was performed. a) Phase contrast image and fluorescence images in cells cultured with or without centrifugation after seeding. b) Fluorescence images in cells cultured with centrifugation after seeding. Fluorescence images show CDF or turo-nor-THCA-24DBD accumulated in bile canaliculi.

Supplementary Table 1. Hepatocyte Maintenance medium

Component		Final Concentration
William's E Medium		
Primary Hepatocyte Maintenance Supplements (CM4000)	A cocktail solution	1/25
	Dexamethasone	0.1μM

Supplementary Table 2. iCell hepatocyte Maintenance medium

Component	Amount (ml)	Final Concentration
RPMI	48	96%
B27	1	2%
Dexamethasone (5 mM)	0.001	0.1 μM
Gentamicin	0.025	25 μg/ml
iCell Hepatocytes 2.0 Medium Supplement	1	1X

Supplementary Table3. TaqMan™ Gene Expression Assay

Gene Symbol	TaqMan Assay ID
CYP1A2	Hs00167927_m1
CYP2C9	Hs00426397_m1
CYP2C19	Hs00426380_m1
CYP2D6	Hs00164385_m1
CYP3A4	Hs00430021_m1
MRP2	Hs00960489_m1
BSEP	Hs00994811_m1
OATP1B1	Hs00272374_m1
NTCP	Hs00161820_m1

Supplementary Table4. Relative value of the bile acids compared to Day16

	Relative value to Day16			
	Day7	Day11	Day14	Day16
Cholic acid	0.03 ± 0.00	0.03 ± 0.00	0.54 ± 0.05	1.00 ± 0.15
Chenodeoxycholic acid	0.17 ± 0.05	0.16 ± 0.03	0.95 ± 0.08	1.00 ± 0.06
Deoxycholic acid	0.98 ± 0.09	1.03 ± 0.04	0.91 ± 0.14	1.00 ± 0.06
Ursodeoxycholic acid	n.d.	n.d.	0.48 ± 0.06	1.00 ± 0.04
Glycochenodeoxycholic acid	0.05 ± 0.01	0.06 ± 0.01	0.61 ± 0.04	1.00 ± 0.02
Glycocholic acid	0.00 ± 0.00	0.00 ± 0.00	0.41 ± 0.04	1.00 ± 0.07
Glycodeoxycholic acid	0.34 ± 0.06	1.04 ± 0.28	5.37 ± 0.94	1.00 ± 0.19
Glycoursodeoxycholic acid	0.20 ± 0.06	0.19 ± 0.01	0.55 ± 0.04	1.00 ± 0.05
Taurochenodeoxycholic acid	1.00 ± 0.05	0.55 ± 0.04	2.02 ± 0.28	1.00 ± 0.16
Taurocholic acid	0.03 ± 0.00	0.02 ± 0.00	0.27 ± 0.14	1.00 ± 1.59
Taurolithocholic acid	1.30 ± 0.30	1.18 ± 0.36	1.85 ± 0.14	1.00 ± 0.31

n.d.: not detected
